# Snack consumption patterns and their associations with risk of incident metabolic syndrome: Tehran lipid and glucose study

**DOI:** 10.1186/s12986-023-00745-0

**Published:** 2023-04-26

**Authors:** Zahra Gaeini, Hanieh Malmir, Parvin Mirmiran, Zahra Feizy, Fereidoun Azizi

**Affiliations:** 1 Nutrition and Endocrine Research Center, Research Institute for Endocrine Sciences, Shahid Beheshti University of Medical Sciences, Tehran, Iran; 2 Department of Nutritional Sciences, Texas Tech University, Lubbock, TX 79409 USA; 3 Endocrine Research Center, Research Institute for Endocrine Sciences, Shahid Beheshti University of Medical Sciences, Tehran, Iran

**Keywords:** Snack, Snack patterns, Metabolic syndrome, Caffeine

## Abstract

**Aim:**

Few studies considered the association between snack patterns and metabolic abnormalities. Here we aimed to characterize the major snack patterns among Iranian adults and determine their association with the risk of metabolic syndrome (MetS).

**Methods:**

This study was conducted on 1713 MetS-free adults who participated in the third phase of the Tehran Lipid and Glucose Study (TLGS). At baseline, dietary intake of snack was assessed using a validated 168-items food frequency questionnaire, and snack patterns were obtained by principal component analysis (PCA). Adjusted Hazard Ratios (HRs) and 95% confidence intervals (CIs) were calculated for the association of incident MetS with the extracted snack patterns.

**Results:**

PCA identified five major snack patterns, defined as “healthy pattern”, “low-fructose pattern”, “high-trans pattern”, “high-caffeine pattern” and “high-fructose pattern”. Participants in the highest tertile of the “high-caffeine pattern” had lower risk of MetS (HR = 0.80, 95% CI = 0.65–0.99, *P* for trend = 0.032). Other snack patterns have not shown any significant association with MetS incidence.

**Conclusions:**

Our findings suggest that consuming a snack pattern with high loads of caffeine, defined as **“**High-caffeine pattern” in the present study, could reduce the risk of MetS in healthy adults. Further prospective studies are needed to more fully determine the association between snack patterns and MetS incidence.

## Introduction

Metabolic syndrome (MetS) is a complex disorder that represents a combination of cardio-metabolic risk factors, including abdominal obesity, dyslipidemia, impaired glucose homeostasis, and hypertension (HTN) [1]. The risk of death from cardiovascular disease and mortality due to other causes is significantly higher in people with MetS [2]. Among several known risk factors for MetS, dietary factors are major modifiable factors [3]. Snacks, i.e., the foods that are not consumed as part of the main daily meals, are important parameters in the diet that are often overlooked [4]. Today, snack consumption is a common feature of the diet. Previous findings indicated that people around the world receive about 22% of their total daily energy intake from snack [5, 6]. Numerous observational studies and clinical trials investigated the association between individual snacks, as separate exposures, and risk of metabolic disorders; they showed that daily consumption of healthy snack, including fruits, vegetables, nuts, and milk, was associated with a lower risk of MetS, while daily intake of cookies, biscuit and sweetened beverages was related to a higher risk of MetS [7–10]. However, it should be considered that such studies may have missed the synergistic or additive effects of intake of multiple snacks. Analyzing the patterns of consumed snacks could dissolve the complexities by uncovering inter-relation of snacks. A number of previous studies have investigated snack patterns and their association with metabolic disorders. A prospective cohort study in Spain revealed a positive association between adherence to an ‘unhealthy snacking pattern’ (characterized by the presence of processed meat products, industrial bakery products and other processed foods like French fries, pizza and fruit syrup) and incidence of MetS [11]. The “dairy and sugar pattern” in a study was associated with a higher risk of elevated glycated hemoglobin and insulin resistance [12]. Another study showed an inverse association between “milk desserts pattern” and values of waist circumference (WC) [13]. Also, “fruit-snack after breakfast”, “fruit-lunch”, “vegetable-dinner”, and “dairy-snack after dinner” were associated with lower mortality risks of cardio-vascular disease (CVD), cancer, and all-cause; whereas “starchy-snack after main meals” was associated with greater risk of CVD and all-cause mortalities [14].

Considering the limited and inconsistent data regarding the association between snack consumption patterns and risk of metabolic abnormalities, we aimed to characterize snack consumption patterns in an adult Asian population, and examine the potential association between snack patterns and risk of MetS.

## Methods

### Study population

**Fig. 1:**
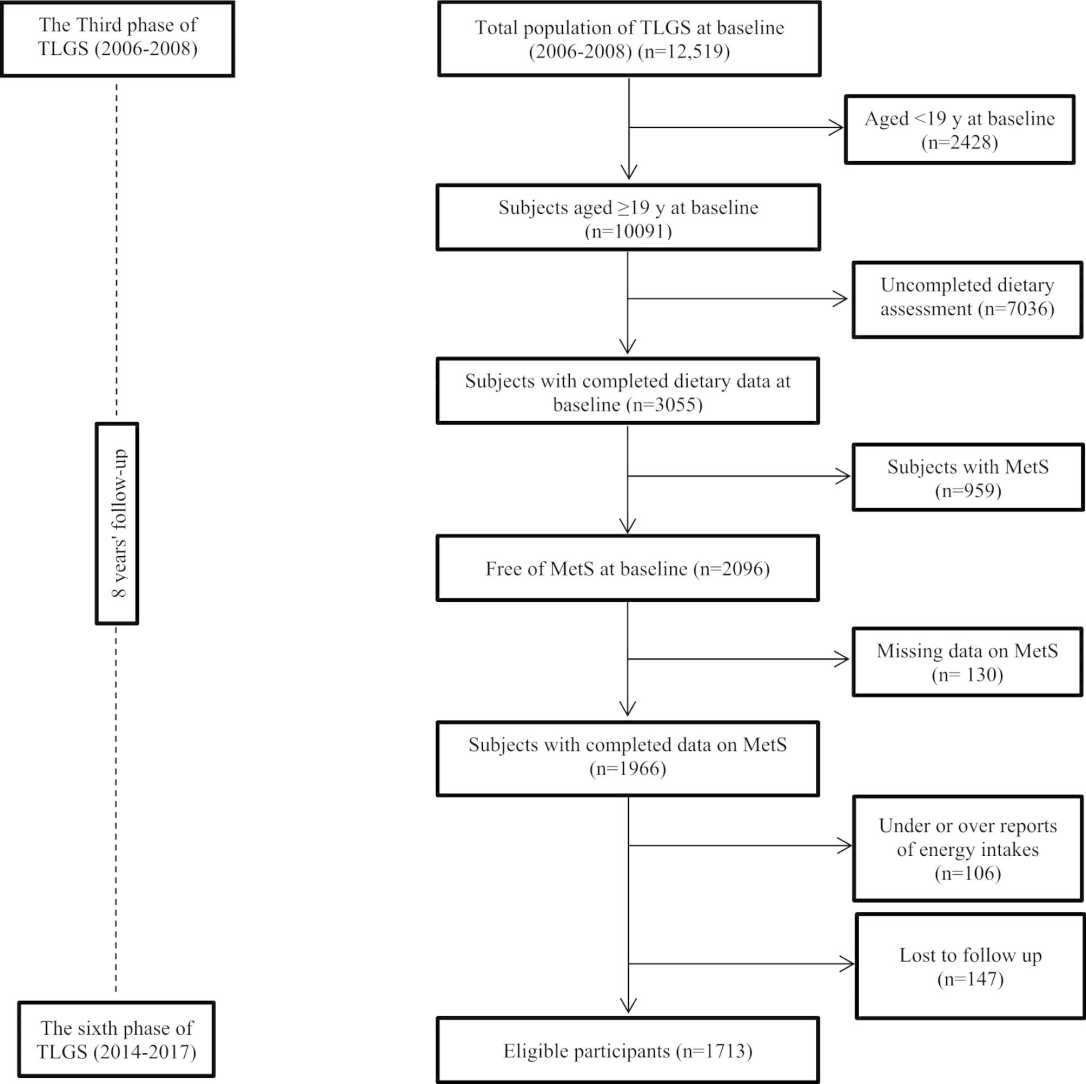
Flow chart of the study

This study was conducted within the framework of the Tehran Lipid and Glucose Study (TLGS), which is an ongoing prospective cohort study aimed to identify the risk factors for non-communicable diseases. The first examination of TLGS was initiated in 1999, and measurements were repeated every three years [15]. In the third phase of the TLGS (2006–2008), there were 10,091 adults aged ≥ 19 years with a complete medical history and physical examination data. For the current study, we excluded participants who had uncompleted dietary data (*n* = 7036), participants with a history of MetS (*n* = 959), participants who had incomplete data in terms of MetS, anthropometric and biochemical measurement (*n* = 130); 1966 healthy adults remained. Then, we excluded participants with under- or over-reports of energy intake (< 800 kcal/d or > 4200 kcal/d, respectively) [16] (*n* = 106) and participants who lost to follow-up (*n* = 147). Finally, 1713 MetS-free adults were followed up to the sixth phase of the TLGS (2014–2017) **(**Fig. 1**)**. The median (inter-quartile range) of the follow-up period was 7.6 (4.9–9.1) years from baseline.

### Anthropometric and demographic assessments

Body weight of the participants was measured to the nearest of 100 gr using digital scales (Seca, Hamburg, Germany), while subjects were minimally dressed and without shoes. Height of the participants was measured to the nearest of 0.5 cm, in a standing position and without shoes, using a tape meter. Body mass index (BMI) was calculated as weight (kg) divided by the square of height (m^2^). WC was recorded to the nearest of 0.1 cm, using a soft measuring tape at the umbilicus and without any pressure on the body surface. Systolic (SBP) and diastolic (DBP) blood pressures were measured using a standard mercury sphygmomanometer calibrated by the Iranian Institute of Standards and Industrial Researches [17]. Before measuring SBP and DBP, the participants remained seated for 15 min.

The Persian version of the modifiable activity questionnaire (MAQ), which was previously validated for participants of TLGS [18], was used for assessing usual physical activity levels. Participants were asked to report the frequency and time spent on their activities of light, moderate, hard, and very hard intensity during the past 12 months, according to a list of common activities of daily life. Physical activity levels were expressed as metabolic equivalent minutes per week (METs-min/week) [19]. Scores ≤ 600 METs-min/week were considered as low physical activity, and scores > 600 METs-min/week were considered as moderate and high physical activity.

### Biochemical measurements

Blood samples were taken from participants after overnight fasting between 7:00 and 9:00 AM. Serum triglyceride (TG), fasting serum glucose (FSG), and 2-hour serum glucose (2 h-SG) levels were measured using an enzymatic colorimetric method, with glycerol phosphate oxidase and glucose oxidase, respectively. High-density lipoprotein-cholesterol (HDL-C) was measured after precipitation of the Apo-lipoprotein B containing lipoproteins with phosphotungstic acid. All blood analysis was done at the research laboratory of the TLGS, using Pars Azmoon kits (Pars Azmoon Inc., Tehran, Iran) and a Selectra 2 auto-analyzer (Vital Scientific, Spankeren, The Netherlands). Both inter- and intra-assay coefficients of variation (CV) at baseline and follow-up phase were less than 5%.

### Dietary assessment

The usual dietary intakes of participants were assessed using a valid and reliable semi-quantitative 168-item food frequency questionnaire (FFQ) at baseline. The reliability and validity of the FFQ have been previously reported [20]. During face-to-face interviews by trained dieticians, participants were asked to report their intake frequency for each food item consumed during the past year on a daily, weekly, or monthly basis. The frequencies were then converted to daily intakes, and portion sizes, reported in household measures, were converted to grams [21]. The USDA food composition table was used to obtain the energy and nutrient content of foods and beverages.

The FFQ items for snacks included biscuits, crackers, cakes, cookies, milk, ice cream, some types of vegetables (cucumber, raw carrot, turnip), all types of fruits, dried fruits, natural fruit juices, canned fruits, nuts, sugar and sugar cube, honey, rock candy, soft drinks, some types of sweets, candies, chocolates, potato chips, puff snacks, doughnut, crème caramel, tea, and coffee.

### Definition of terms and outcomes

MetS was defined as having at least three of the following metabolic abnormalities [22, 23]: 1-Hyperglycemia (FSG ≥ 100 mg/dL (5.6 mmol/L) or self-reported taking of blood glucose-lowering medication); 2-Hypertriglyceridemia (serum TG ≥ 150 mg/dL (1.69 mmol/L) or using lipid-lowering drugs); 3-Low HDL-c (serum HDL-c < 40 mg/dL (1.04 mmol/L) for men and < 50 mg/dL (1.29 mmol/L) for women, or drug treatment); 4-HTN (SBP/DBP ≥ 130/85 mm Hg or drug treatment for HTN), and 5-Abdominal obesity (WC ≥ 95 cm for both genders). For WC, we used the modified cutoff points for Iranian adults [24].

### Statistical analyses

Principle component analysis (PCA) was used to determine patterns of dietary snacks, based on the 15 main snack groups (fruits, vegetables, dried fruits, milk, cake and cookies, sweets, soft drink, nuts, sugars, chocolates, tea and coffee, desserts, salty snacks, added sugar fruits, fruit juices), with varimax rotation and correlation matrix at baseline. All 15 snack groups contributed to the pattern score calculation; however, snacks with an absolute component loading score of ≥ 0.40 and < -0.40 were selected to describe the patterns. The Kaiser-Meyer-Olkin statistic, a measure of sampling adequacy, was 0.62, and the *P*-value for Bartlett’s test of sphericity was < 0.001. The factor scores for each extracted pattern were calculated using the sum of the frequency of consumption multiplied by factor loadings on each snack pattern. We identified five major patterns based on the scree plot (eigenvalue > 1) and categorized them into tertiles.

Baseline characteristics of participants as mean (± SD) values for continuous variables and frequencies (%) for categorical variables were compared according to the tertiles of snack patterns scores using ANCOVA. The incidence of MetS over the follow-up period was considered as a dichotomous variable (yes/no) in the models.

Cox proportional hazards regression models with person-years as the underlying time metric were used to estimate hazard ratios (HRs) and 95% confidence intervals (CIs) for the association between each snack pattern and MetS incidence. Time to event for MetS was defined as time to end of follow-up (censored cases) or time to having an event, whichever occurred first. The proportional hazard assumption of the multivariable Cox model was assessed using Schoenfeld’s global test of residuals.

We performed univariate analysis to obtain the final multivariable models and determine confounding variables. Variables with P_E_ less than 0.2 in the univariate analyses were selected as potential confounders. Confounders adjusted in the Cox models, included sex (men/women), age (years), BMI (kg/m^2^), smoking (yes/no), physical activity level (low/high), total energy (kcal/d) and protein (g/d) intake.

All statistical analyses were performed using the Statistical Package for Social Science (version 20; IBM Corp., Armonk, NY, USA), *P*-values < 0.05 being considered significant.

## Results

The mean (± SD) age of the participants was 36.99 (± 13.12) years, and 40.9% of them were men. The median (inter-quartile range) of follow-up duration was 7.6 (4.9–9.1) years; the incidence rate of MetS during that time was 34.8%.

The mean (± SD) of percentage of energy intake from each snack group was 7.48 (± 5.46) for fruits, 0.60 (± 0.63) for vegetables, 1.43 (± 1.95) for dried fruits, 0.40 (± 0.71) for natural fruit juices, 3.59 (± 3.73) for milk, 2.31 (± 2.98) for biscuits, crackers, cakes and cookies, 1.72 (± 2.01) for sweets, 1.67 (± 2.33) for nuts, 3.42 (± 3.34) for sugars, 0.35 (± 0.57) for chocolates, 0.29 (± 0.26) for coffee and tea, 1.13 (± 1.97) for desserts, 1.80 (± 2.70) for salty snacks, 0.26 (± 0.71) for added sugar fruits, 0.06 (± 0.12) for soft drinks.

**Fig. 2:**
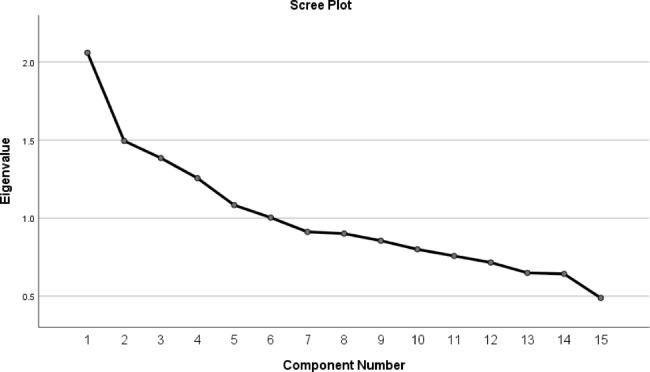
Scree plot for extraction of snack patterns by principal component analysis. The 15 snacks were used as input variables and snack patterns based on eigenvalues > 1 were identified as main snack patterns

The factor loading matrix of 15 main snack groups and variances of each of five snack patterns are shown in Table 1. PCA identified five major snack patterns **(**Fig. 2**)**, which explained 48.5% of the total variation of 15 snack groups. The first pattern is characterized by high loads of nuts, fruits, dried fruits, vegetables, milk, and chocolate. The second pattern had a high negative correlation with the intake of soft drinks and milk. Pattern 3, characterized by high loads of cake and cookies and salty snacks, had a negative correlation with dried fruits. Coffee, tea and sugar were highly loaded in the fourth pattern. The fifth pattern positively correlated with fruit juice, desserts, and added sugar fruits. The patterns were defined as “healthy pattern”, “low-fructose pattern”, “high-trans pattern”, “high-caffeine pattern” and “high-fructose pattern”, respectively.

**Table 1:** Factor loading matrix and explained variances for major snack patterns identified by factor analysis ^1^

Snacks	Patterns				
	1	2	3	4	5
Nuts	**0.444**				-0.311
Fruits	**0.594**		-0.372		
Dried fruits	**0.525**		**-0.433**		
Vegetables	**0.431**				
Fruit juice	0.377				**0.437**
Cake & cookies			**0.429**		
Soft drink		**-0.681**		0.321	
Coffee & tea			-0.328	**0.603**	
Milk	**0.407**	**-0.627**			
Desserts					**0.480**
Sugar		0.323		**0.625**	
Sweets	0.384	0.375	0.338		
Chocolate	**0.428**	0.366			
Salty snacks		0.301	**0.558**		
Added sugar fruits	0.302				**0.649**
Explained variance (%)	13.72	9.96	9.23	8.37	7.22
Cumulative explained variance (%)	13.18	23.69	32.92	41.29	48.51

^1^ Principle Component Analysis (PCA) performed on 15 snack group. Snacks with loadings > 0.40 and less than − 0.40 (in bold) are being characteristic for the five patterns; loadings less than 0.3 (in absolute value) are suppressed

General characteristics of participants based on tertiles of snack patterns are shown in Table 2. There was no significant difference in general characteristics of participants across tertile categories of the “healthy pattern”. Across tertile categories of the “low-fructose pattern”, mean serum HDL-c levels increased, while male percentage and smokers’ percentage decreased (*P*-value < 0.05). Mean age and BMI increased significantly across tertile categories of the “high-trans pattern”, while the male percentage and the percentage of participants with a low physical activity level decreased (*P*-value < 0.05). Across tertile categories of the “high-caffeine pattern”, mean levels of TG, cholesterol, and LDL-c decreased significantly (*P*-value < 0.05). Finally, the mean age, percentage of smokers, and levels of SBP and DBP increased significantly across tertile categories of the “high-fructose pattern”, while the percentage of participants with a low physical activity level decreased (*P*-value < 0.05).

**Table 2:** Baseline characteristics of participants based on the snack patterns

Baseline characteristics	Healthy pattern			Low-fructose pattern			High-trans pattern			High-caffeine pattern			High-fructose pattern		
	T1		T3	T1		T3	T1		T3	T1		T3	T1	T3	
Age, year	36.85 ± 12.84	36.78 ± 13.45		37.78 ± 13.18	36.46 ± 13.05			35.37 ± 12.56	38.32 ± 13.33*	37.36 ± 12.85	36.60 ± 13.24		35.66 ± 12.15		36.90 ± 13.54*
Male, %	39.8	42.0		45.5	35.2*			46.2	37.0*	43.8	39.2		40.3		40.8
BMI, kg/m^2^	25.73 ± 4.61	25.59 ± 4.53		25.48 ± 4.49	26.05 ± 4.60			25.40 ± 4.18	26.14 ± 4.70*	25.77 ± 4.21	25.65 ± 4.55		25.50 ± 4.29		25.71 ± 4.57
WC, cm	85.15 ± 12.40	84.94 ± 12.25		85.85 ± 12.17	85.00 ± 12.29			85.08 ± 12.26	85.74 ± 12.19	85.52 ± 11.91	84.71 ± 12.32		84.59 ± 11.46		85.29 ± 12.74
Current smoker, %	7.6	7.6		11.7	5.4*			8.6	7.1	8.9	6.2		8.0		10.6*
Low physical activity *, %	40.3	39.2		40.0	36.7			42.6	34.8*	41.0	38.6		41.6		40.3*
SBP, mmHg	107.1 ± 13.23	106.8 ± 14.25		108.2 ± 14.05	106.4 ± 12.89			107.2 ± 13.57	107.9 ± 14.18	106.9 ± 14.36	107.1 ± 13.60		105.8 ± 13.23		107.4 ± 13.38*
DBP, mmHg	70.98 ± 9.06	70.43 ± 9.77		71.25 ± 10.35	70.61 ± 9.04			70.80 ± 10.03	71.09 ± 9.33	71.08 ± 9.73	70.40 ± 9.15		69.72 ± 9.72		71.30 ± 9.29*
Serum TG, mg/dL	107.7 ± 49.22	109.0 ± 53.10		111.0 ± 51.27	107.1 ± 50.74			109.9 ± 51.15	110.1 ± 49.51	114.7 ± 53.29	103.2 ± 49.43*		105.6 ± 45.45		110.0 ± 55.82
Serum cholesterol, mg/dL	179.0 ± 36.11	177.9 ± 36.75		179.0 ± 36.74	180.2 ± 36.86			176.2 ± 36.81	181.0 ± 37.19	183.3 ± 37.45	175.7 ± 36.79*		176.5 ± 36.27		180.8 ± 39.06
Serum LDL-c, mg/dL	111.7 ± 31.78	111.1 ± 31.66		112.1 ± 32.39	113.0 ± 31.75			109.9 ± 32.16	113.3 ± 32.02	115.1 ± 31.94	109.7 ± 32.23*		110.6 ± 31.93		113.6 ± 33.42
Serum HDL-c, mg/dL	45.78 ± 10.46	44.87 ± 10.03		44.48 ± 9.76	45.91 ± 10.11*			44.41 ± 10.08	45.70 ± 10.19	45.29 ± 10.76	45.22 ± 9.76		44.89 ± 9.97		45.25 ± 10.17
FSG, mg/dL	85.53 ± 9.18	86.00 ± 11.18		86.43 ± 12.58	85.36 ± 9.96			85.66 ± 9.99	85.89 ± 12.52	85.35 ± 9.90	86.16 ± 13.27		85.87 ± 10.43		85.77 ± 8.78

BMI; body mass index, WC; waist circumference, SBP; systolic blood pressure, DBP; diastolic blood pressure, TG; triglyceride, LDL-c; low density lipoprotein cholesterol, HDL-c; high density lipoprotein cholesterol, FSG; fasting serum glucose

Data represented as mean ± SD and percent

* *P*-value < 0.05

Dietary intakes of participants across tertile categories of snack patterns are presented in Table 3. Total energy intake of participants increased across tertile categories of all five snack patterns (*P*-value < 0.05). There was no significant difference in dietary intakes of macronutrients of participants between tertile categories of the “healthy pattern”. Across tertile categories of the “low-fructose pattern”, dietary intakes of total protein, animal protein, total fat, and saturated fatty acids (SFA) increased, and dietary intakes of carbohydrates, plant proteins, and poly-unsaturated fatty acids (PUFA) decreased (*P*-value < 0.05). With increasing tertiles of the “high-trans pattern”, carbohydrate intake increased; however, dietary intake of total fat, monounsaturated fatty acids (MUFA) and polyunsaturated fatty acids (PUFA) decreased (*P*-value < 0.05). Across tertile categories of the “high-caffeine pattern”, we observed a decrease in plant protein intake (*P*-value < 0.05). Finally, dietary intake of animal protein and total fat increased across tertile categories of the “high-fructose pattern”, while dietary intake of carbohydrate and plant protein decreased (*P*-value < 0.05).

**Table 3:** Dietary intakes of participants based on the snack patterns

Dietary intakes	Healthy pattern		Low-fructose pattern		High-trans pattern		High-caffeine pattern		High-fructose pattern	
	T1	T3	T1	T3	T1	T3	T1	T3	T1	T3
Total energy, kcal/d	2322 ± 679.6	2366 ± 767.7*	2104 ± 711.2	2405 ± 690.7*	2178 ± 692.5	2526 ± 714.9*	2236 ± 678.8	2458 ± 734.2*	2283 ± 695.1	2432 ± 732.4*
Carbohydrates, en%	57.16 ± 6.94	57.61 ± 7.75	58.00 ± 7.50	56.13 ± 6.89*	56.30 ± 7.06	58.48 ± 7.20*	56.97 ± 6.80	57.51 ± 7.53	57.05 ± 7.17	56.71 ± 7.38*
Total proteins, en%	13.72 ± 2.41	13.59 ± 2.50	13.26 ± 2.38	14.27 ± 2.44*	13.71 ± 2.36	13.58 ± 2.43	13.74 ± 2.29	13.65 ± 2.43	13.67 ± 2.31	13.59 ± 2.33
Animal protein, en%	4.66 ± 2.07	4.63 ± 2.27	3.53 ± 1.52	6.10 ± 2.15*	4.63 ± 2.05	4.73 ± 2.14	4.56 ± 1.92	4.73 ± 2.15	4.58 ± 1.92	4.87 ± 2.27*
Plant protein, en%	7.51 ± 1.56	7.55 ± 1.70	7.86 ± 1.70	7.07 ± 1.47*	7.49 ± 1.57	7.45 ± 1.54	7.62 ± 1.52	7.24 ± 1.61*	7.51 ± 1.52	7.21 ± 1.56*
Total fat, en%	31.62 ± 6.75	31.58 ± 7.47	30.97 ± 7.42	32.31 ± 6.57*	32.21 ± 7.18	31.08 ± 6.72*	31.73 ± 6.65	31.75 ± 7.15	31.78 ± 7.01	32.31 ± 7.16*
SFA, en%	10.71 ± 2.79	10.89 ± 8.44	9.97 ± 2.89	11.65 ± 8.27*	10.84 ± 2.99	10.51 ± 3.61	10.94 ± 8.27	10.61 ± 3.61	10.70 ± 3.06	10.96 ± 3.55
MUFA, en%	10.96 ± 2.79	10.93 ± 2.95	10.94 ± 3.07	10.99 ± 2.62	11.23 ± 2.97	10.69 ± 2.70*	11.04 ± 2.71	10.91 ± 2.85	11.04 ± 2.79	10.14 ± 2.92
PUFA, en%	6.49 ± 2.23	6.59 ± 2.36	6.80 ± 2.52	6.34 ± 2.10*	6.73 ± 2.43	6.32 ± 2.05*	6.67 ± 2.19	6.62 ± 2.32	6.61 ± 2.58	6.61 ± 2.31

SFA; saturated fatty acids, MUFA; mono-unsaturated fatty acids, PUFA; poly-unsaturated fatty acids

Data represented as mean ± SD and percent

* *P*-value < 0.05

HRs (95% CIs) of MetS in relation to five snack patterns scores are shown in Table 4. Participants in the highest tertile of the “high-caffeine pattern” score had lower risk of MetS in the crude (HR = 0.77, 95% CI = 0.63–0.94, *P* for trend = 0.009) and adjusted (HR = 0.80, 95% CI = 0.65–0.99, *P* for trend = 0.032) models. There was no significant association between MetS incidence and other snack pattern scores.

**Table 4:** Hazard ratio (95% confidence interval) of metabolic syndrome across tertiles of snack patterns

	Tertiles of snack patterns			*P* for trend
	1	2	3	
**Healthy pattern**				
*Cases/ person-year*	*204/3819*	*207/3712*	*185/3778*	
HR (95% CI) Crude Model	1.00	1.04 (0.86–1.27)	0.94 (0.77–1.15)	0.568
HR (95% CI) Model 1	1.00	1.05 (0.86–1.28)	0.99 (0.81–1.21)	0.939
HR (95% CI) Model 2	1.00	1.06 (0.87–1.30)	0.99 (0.81–1.21)	0.938
**Low-fructose pattern**				
*Cases/ person-year*	*210/3677*	*203/3789*	*183/3843*	
HR (95% CI) Crude Model	1.00	0.93 (0.77–1.13)	0.82 (0.67-1.00)	0.054
HR (95% CI) Model 1	1.00	0.98 (0.81–1.20)	0.85 (0.69–1.04)	0.119
HR (95% CI) Model 2	1.00	0.97 (0.80–1.18)	0.84 (0.68–1.04)	0.114
**High-trans pattern**				
*Cases/ person-year*	*196/3765*	*195/3836*	*205/3709*	
HR (95% CI) Crude Model	1.00	0.97 (0.79–1.18)	1.06 (0.87–1.29)	0.565
HR (95% CI) Model 1	1.00	0.92 (0.75–1.12)	0.96 (0.79–1.18)	0.724
HR (95% CI) Model 2	1.00	0.92 (0.75–1.12)	0.94 (0.77–1.16)	0.578
**High-caffeine pattern**				
*Cases/ person-year*	*225/3687*	*196/3845*	*175/3777*	
HR (95% CI) Crude Model	1.00	0.84 (0.69–1.03)	0.77 (0.63–0.94)	0.009
HR (95% CI) Model 1	1.00	0.85 (0.70–1.03)	0.82 (0.67–0.99)	0.048
HR (95% CI) Model 2	1.00	0.85 (0.70–1.03)	0.80 (0.65–0.99)	0.032
**High-fructose pattern**				
*Cases/ person-year*	*193/3845*	*207/3696*	*196/3768*	
HR (95% CI) Crude Model	1.00	1.11 (0.91–1.36)	1.03 (0.85–1.27)	0.736
HR (95% CI) Model 1	1.00	1.00 (0.82–1.22)	1.00 (0.82–1.22)	0.999
HR (95% CI) Model 2	1.00	1.01 (0.82–1.24)	0.99 (0.80–1.21)	0.907

Data are hazard ratio (95% confidence interval); proportional hazard Cox regression was used. HR, Hazard Ratio; CI, confidence interval

Model 1 adjusted for sex (men/women), age (years), body mass index (kg/m^2^), smoking (yes/no), physical activity level

Model 2 additionally adjusted for dietary intake of total energy (kcal/d) and total protein (g/d)

## Discussion

In this prospective cohort study among participants of the TLGS, we identified five snack patterns using PCA, including “healthy pattern”, “low-fructose pattern”, “high-trans pattern”, “high-caffeine pattern” and “high-fructose pattern”. We found that the “high-caffeine pattern” was associated with a 20% lower risk of MetS. Other snack patterns have not shown any significant association with MetS incidence.

Most of the previous studies investigating the association between caffeine and coffee consumption and risk of MetS are in line with our findings. A previous meta-analysis of 13 observational studies indicated that the highest level of coffee consumption, compared to the lowest intakes, decreases the risk of MetS by 13%. Non-linear dose-response meta-analysis of this study showed that a higher intake of coffee (at the levels of 0.5 to 2.5 cups per day) was associated with a reduced risk of MetS (P non-linearity < 0.001) [25]. In a cohort study of 15,691 women, the highest coffee consumption quartile exhibited 25% lower odds of suffering from MetS compared to those in the control group (OR = 0.75; 95% CI = 0.67–0.84; P for trend < 0.001) [26]. A cross-sectional study in Poland revealed that moderate drinkers of coffee had 17% lower odds of MetS compared with non-drinkers (OR = 0.83, 95%CI = 0.72–0.97). However, tea consumption was not related to MetS in general [27]. Also, coffee consumption was related to lower blood pressure and HDL-c concentrations, and tea consumption was related to lower values of WC. Moreover, a 16-weeks clinical trial in rats has shown that treatment with caffeine in the rats fed the high-carbohydrate, high-fat diet decreased body fat and SBP, improved glucose tolerance and insulin sensitivity, and attenuated cardiovascular and hepatic abnormalities [28].

A cross-sectional study of 3284 adults suggested that increasing coffee consumption was linked to the improved status of MetS and its component, including hypertriglyceridemia and hypertension [29]. Moreover, greater coffee consumption was associated with 47% lower risk of hypertriglyceridemia in Japanese adults [30]. The hypothesized biological mechanisms for beneficial effects of caffeine include inhibiting critical enzymes and increasing the level of cAMP, so inhibiting lipolysis and hydrolysis of TG to glycerol and free fatty acids [31]. Also, it was shown that caffeine could reduce intestinal glucose absorption through inhibition of glucose-6-phosphate translocase 1. Moreover, the hepatic output of glucose decreased and insulin sensitivity improved due to the magnesium content of coffee [32]. In addition, caffeine can regulate adenosine pathway and activate beta-adrenergic receptors [33].

It is notable that factors such as the type of tea or coffee consumed (caffeinated coffee, decaffeinated coffee, or instant coffee, green tea, or black tea), methods of preparation and processing of them, genetic backgrounds, daily amounts of coffee or tea consumption and habitual consumption of sweets or sugar accompanied by tea or coffee, could affect the results. As we reported in the present study, the “high-caffeine pattern” also had high loads of sugar intake. The beneficial effects of the “high-caffeine pattern” observed in the present study are obviously due to the caffeine and other antioxidants and polyphenols of coffee and tea, not sugar, and health recommendations must be considered in limiting the sugar and sweets consumption along with coffee and tea.

Although neither the “low-fructose pattern” nor the “high-fructose pattern” had no significant association with MetS incidence in our study, there are numerous studies that investigated the association between fructose intake and metabolic health. Previous studies have showed different effect of fructose based on its source; fructose from sweetened beverages or added fructose snacks has different effect compared to fructose from natural sources such as fruits. Findings from Framingham cohort study indicated that greater consumption of soft drinks and added-fructose beverages were associated with increased risks of MetS, abdominal obesity, impaired fasting glucose, higher blood pressure, hypertriglyceridemia, and low HDL-c [34]. In a randomized controlled trial on 74 adult men, consumption of 200gr additional fructose resulted in increased levels of SBP and DBP and a higher prevalence of hyperglycemia and hyperlipidemia [35]. Also, a recent meta-analysis of animal studies indicated that consuming fructose-rich beverages leads to increased body weight, elevated SBP, hyperglycemia, hyperinsulinemia, and hypertriglyceridemia [36]. Fructose can lead to an increased level of lipid profiles, insulin resistance, and leptin resistance, disturbing body fat regulation and contributing to obesity [37, 38]. High fructose also results in higher uric acid levels, a byproduct of fructose metabolism, and is linked to endothelial dysfunction and HTN [39, 40]. High fructose consumption could affect blood pressure via up-regulating sodium and chloride transporters, resulting in a state of salt overload. It also activates vasoconstrictors, inactivates vasodilators, and over-stimulates the sympathetic nervous system [39].

In the present study, adherence to the “healthy pattern” indicated no association with MetS. Previous publications reported beneficial effects of adherence of a healthy diet with high levels of fruit and vegetables, milk, and nuts on metabolic status [41–43]. On the other hand, adherence to an ‘unhealthy snacking pattern’ (characterized by the presence of processed meat products, industrial bakery products and other processed foods like French fries, pizza and fruit syrup) was associated with a higher risk of MetS in a previous cohort study [11]. On the other hand, we found no significant association between the “high-trans pattern” and risk of MetS. Finding no association in the present study could be the result of overlapping effects of food consumption on dietary patterns. The general dietary pattern and eating habits are various in different societies, and it should be considered in determining snack effects as part of the overall diet. Often, the amount of snacks, time of snack consumption, and the number of snacks servings vary from person to person. The effects of food intake should also be interpreted in interaction with the genome and other environmental exposures. Recent findings of nutrigenomics research indicated that the effects of foods and dietary patterns without considering various individuals’ genome could not be comprehensive.

To the best of our knowledge, there is limited data regarding the relationship between snack patterns and MetS. The prospective design and long follow-up period are strength points of the current study. In addition, participants of the present study represent the general population and all assessment processes, including interview and questionnaire, have acceptable validity and reliability. Like all researches, this study has some limitations. Although this was an exploratory analysis, the observed associations were rather minor. Also, since adding many variables to adjust in the models would lead to instability of the models and could reduce the study power, we selected a number of the potential confounding variables as final confounders to adjust for, based on the univariate analysis, so the residual confounders’ effect was not considered. Changes in an individual’s diet and other risk factors during the study follow-up might result in biased estimated HRs. Snack consumption was assessed using FFQ; therefore, measurement errors and overestimation are probable, which could have led to misclassification of individuals into wrong categories and weakened the associations. Also, we did not have any information on the time and number of snacks consumption by participants. Hence, a better interpretation of the results would be obtained if this information was available. Finally, the findings of the study have been obtained using the principal component method of pattern analysis, which is not specific to the study population.

## Conclusion

“High-caffeine pattern” was associated with lower risk of MetS. However, other snack consumption patterns did not show any significant association with MetS. Further observational studies are needed to clarify the associations.

## Data Availability

The datasets used and/or analyzed during the current study available from the corresponding author on reasonable request.

## List of Abbreviations

BMI: Body mass index
CI: Confidence intervals
DBP: Diastolic blood pressure
FFQ: Food frequency questionnaire
HDL-c: High-density lipoprotein cholesterol
HR: Hazard ratios
HTN: Hypertension
MET: Metabolic equivalent
MetS: Metabolic syndrome
SBP: Systolic blood pressure
TLGS: Tehran Lipid and Glucose Study
TG: Triglyceride
